# The Vienna self-assessment questionnaire for hospitals: Three explorative case studies in Belgium

**DOI:** 10.1093/eurpub/ckac129.378

**Published:** 2022-10-25

**Authors:** G Henrard, J Rademakers

**Affiliations:** 1 Département de Médecine Générale, Université Liege, Liege, Belgium; 2 Hoofd Onderzoeksafdeling Nivel, Maastricht University, Maastricht, Netherlands

## Abstract

**Background:**

One way to develop adequate health literacy responsive policy and strategies in hospitals is the use of self-assessment tools to raise awareness, help prioritize action and mobilize stakeholders. In this study we have piloted the French version of the Vienna Health Literate Organisation (V-HLO-fr) tool in three hospitals to explore its feasibility.

**Methods:**

We performed explorative case studies in the three main hospitals of Liège (Belgium). Our mode of application of the V-HLO-fr was inspired by the ‘RAND Appropriateness’ method: first, individual members of an internal multidisciplinary panel filled out the questionnaire and then the results were discussed collectively in each hospital during a ‘round table’ meeting. The feasibility of the process was assessed by direct observation of the round tables and with semi-structured phone interviews

**Results:**

The V-HLO-fr tool was fully applied in the three targeted hospitals and the process seems to be acceptable, practicable and integrable. Its mode of application, formalized by taking inspiration from the RAND method, could be further improved, e.g. by paying more attention to recruiting and supporting participants. Strengths (e.g. the facilitation of patient navigation to the hospital) and weaknesses (e.g. the provision of easy to read, understand and act on health information materials) in terms of health literacy responsiveness have been highlighted.

**Conclusions:**

V-HLO-fr could be a suitable tool to create awareness and formulate targeted actions to further strengthen hospitals health literacy responsiveness.

Those explorative case studies give:

– an overall positive signal about the feasibility of the V-HLO-fr

– useful feedback to further formalize and refine its procedure of application.

